# Low Diastolic Blood Pressure as a Risk for All-Cause Mortality in VA Patients

**DOI:** 10.1155/2013/178780

**Published:** 2013-03-27

**Authors:** Steven Tringali, Charles William Oberer, Jian Huang

**Affiliations:** ^1^Department of Medicine, Veterans Administration Central California Health Care System, 2615 E Clinton Avenue, Fresno, CA 93703, USA; ^2^Department of Medicine, University of California, San Francisco, Fresno Medical Education Program, 155 N Fresno Avenue, Fresno, CA 93701, USA

## Abstract

*Background*. A paradoxical increase in cardiovascular events has been reported with intensively lowering diastolic blood pressure (DBP). This J-curve phenomenon has challenged the aggressive lowering of blood pressure, especially in patients with coronary artery disease. *Objective*. Our objective was to study the effects of low DBP on mortality and determine a threshold for which DBP should not be lowered beyond. *Methods*. We evaluated a two-year cross-section of primary care veteran patients, from 45 to 85 years of age. Receiver operating characteristics (ROC) were employed to establish an optimal cut-off point for DBP. Propensity-score matching and multivariate logistic regression were used to control for confounders. All-cause mortality was the primary outcome. *Results*. 14,270 patients were studied. An ROC curve found a threshold value of DBP 70 mmHg had the greatest association with mortality (*P* < 0.001). 49% of patients had a DBP of 70 mmHg or less. Using a propensity-matched multivariate logistic regression, odds ratio for all-cause mortality in subjects with a DBP less than 70 mmHg was 1.5 (95% CI 1.3–1.8). *Conclusions*. Reduction of DBP below 70 mmHg is associated with increased all-cause mortality. Hypertension guidelines should include a minimum blood pressure target.

## 1. Introduction

Hypertension affects 29% of male and 25% of female adults worldwide [1]. Its impact on mortality has improved with advances in detection and treatment, yet the U.S. mortality rate still lies at 14.3 per 1,000 people per year [2]. Mortality gaps in hypertensive versus nonhypertensive patients persist due to its occurrence with other appendages of the metabolic syndrome, thus treatment advances. As treatment has expanded, so has its intensity. Recently the question has frequently been raised as to potential harms associated with aggressive treatment of hypertension [3]. A systematic review of aggressive versus standard blood pressure targets did not find any benefit in total mortality when blood pressure is lowered less than 140/90 mmHg [4].

The incidence in cardiovascular events, including mortality, increases with extremes in blood pressure. The paradoxical increase in events at lower blood pressures has been represented by a J-shaped or U-shaped curve. The J-curve phenomenon has been researched since 1979 [5] and has been amplified with individual trials, post hoc analyses, and systemic reviews in support of this finding. Not all patients appear to be equally affected by the J-curve, if at all. Patient with established coronary artery disease (CAD) and diabetes are the most affected by an overcorrection of blood pressure [6]. Elevation in systolic blood pressure has been a more important predictor of mortality than diastolic blood pressure (DBP) [7]. On the other hand, low DBP in patients treated for hypertension has been associated with increased risk of cardiovascular disease [8, 9].

This study evaluates the concept of the J-curve in DBP across a large primary care population with a high prevalence of CAD, diabetes, and hypertension. We sought to determine a threshold for which DBP should not be lowered beyond. We also evaluated low DBP as an independent risk factor for all-cause mortality.

## 2. Materials and Methods

### 2.1. Study Design

A cross-sectional study of predominantly male patients at the VA Central California Healthcare System aged from 45 to 85 years was conducted over a 2-year period. Data were collected from the electronic medical record and provided demographic information, vital signs, comorbid diagnoses, and medications. The study was approved by the VA Northern California Health Care System Institutional Review Board.

### 2.2. Patient Population

All patients at least 45 years of age or older with a minimum of one outpatient encounter with recorded blood pressure were included in the study.

### 2.3. Variables

The primary outcome, death from any cause, was used as the dependent variable. Blood pressure (BP) was collected at the time of an ambulatory encounter. Pulse pressure was calculated as systolic BP minus diastolic BP. Covariates were included that were thought to contribute to the overall risk of mortality as well as variables that might alter a patient's blood pressure goals. These included age, body mass index, and the presence of comorbidities including CAD, hypertension, diabetes, cerebrovascular disease, and chronic kidney disease. The investigators felt that these conditions were the most likely to contribute to the cardiovascular causes of death and were important potential confounding variables in this study. CAD was defined by ICD9 code, by a history of myocardial infarction with abnormal electrocardiogram or troponin elevation, coronary artery bypass graft surgery, percutaneous coronary intervention, or coronary stent placement. Data were also gathered on the classes of blood pressure medications used to treat the patients.

### 2.4. Statistical Analysis

Grouping was based on a cut-off point for DBP. Receiver operating characteristic curve was used to determine the optimal cut-off point for DBP as a continuous scale against death. Baseline characteristics were compared between the two groups with the use of the chi-square test and the independent samples *t*-test. Interval likelihood ratios were calculated for each interval for DBP against all-cause mortality along with 95% confidence interval (CI). Multivariate logistic regression (LR) assessed for independence of low DBP as a risk for all-cause mortality.

Propensity score matching was conducted using DBP grouping as above mentioned for the dependent variable. Each study subject received a propensity score based on the presence of selected covariates. Covariates included age, comorbidities including CAD, hypertension, diabetes, chronic kidney disease, cerebrovascular disease, total number of antihypertensive medications, and individual medication classes. Finally, subjects were matched based on their scores, looking for the closest match.

Statistical analyses were carried out with SPSS Statistics Software for Windows, Version 20.0 (IBM Corp., Armonk, NY). Propensity score matching was carried out using R Project for Statistical Computing, Version 2.12.0 (R Development Core Team, Vienna, Austria) along with the SPSS R Essentials plug-in.

## 3. Results

A total of 14,270 patients were included in the study. The mean age of study population was 67 years with 96% male patients. The prevalence of comorbid conditions was as follows: hypertension 66.7%, diabetes 29.5%, CAD 19.5%, stroke 9.3%, and chronic kidney disease 6.9%.

**Figure 1:**
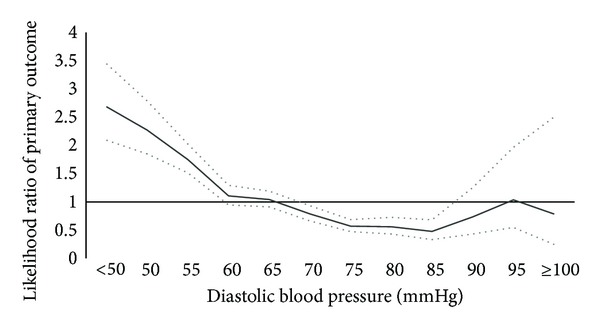
Interval likelihood ratios of all-cause mortality against a range of diastolic blood pressure. (Upper and lower 95% confidence intervals denoted by dotted lines.)

Interval likelihood ratios of all-cause mortality as a function of DBP were calculated and are shown graphically (Figure 1). The lower 95% CI was greater than one for values of a DBP ranging 55 mmHg and less. The only values in the interval likelihood ratio that achieved a 95% CI less than one were DBP ranges from 70 to 85 mmHg. Those values greater than 85 mmHg did not achieve statistical significance. Eighteen percent of patients had an ambulatory DBP of 60 mmHg or below, while 49% had a reading of 70 mmHg or less.

A receiver operating characteristic curve found that the threshold value of DBP with the greatest specificity and sensitivity for mortality was 70 mmHg (*P* < 0.001). Patients were grouped according to DBP less than 70 mmHg and those at or above 70. Baseline characteristics of these two groups were significantly different with respect to age, comorbidities, and use of antihypertensive medications (Table 1). To adjust for such differences, all subjects were assigned a propensity score and then matched based on that score. The results were a closely paired group of 8856 patients with small differences between the groups.

After matching, the comorbidities were similar in the two groups with the exceptions of diabetes (31.7% versus 29.7%, *P*  0.04) and CAD (22.2% versus 18.8%, *P* < 0.001), with more patients having diabetes and CAD in the group with lower DBP. Medication use by class was similar in both groups except for a trend in increased use of loop diuretics in the low DBP group (7.5% versus 6.4%, *P*  0.037). There was no difference in the total number of antihypertensive medications in the two groups (Table 2). A multivariate logistic regression was used to adjust for low DBP, pulse pressure, and CAD as potential confounders and assess for independence in their association with all-cause mortality. Other recorded comorbidities were also included. Among the comorbid conditions, low DBP, along with pulse pressure, CAD, chronic kidney disease, and cerebrovascular disease were each associated with all-cause mortality after controlling for each comorbid condition. Neither diabetes nor hypertension achieved statistical significance (Table 3). The odds ratio for all-cause mortality in subjects with a DBP less than 70 mmHg was 1.34 (95% CI 1.11–1.61) (Table 3).

## 4. Discussion

This study of primary care veterans offers additional insight into the relationship between low-DBP and mortality. This study utilizes a large sample size to evaluate the effects of the J-curve in DBP and to define a lower threshold for DBP. In our study population, the risk of death at various diastolic pressures was not continuous but followed the J-shared curve that has been established previously [10].

The majority of studies looking at harm with aggressive BP lowering have not been consistent in defining a limit to which BP should not be lowered beyond. The data in this study suggest that the benefit of lower DBP is limited to the range of 70–85 mmHg, with a nonstatistically significant trend between 60–65 mmHg. Any DBP value less than 60 mmHg increases the likelihood of all-cause mortality.

When assessing comorbid conditions for confounding biases, the multivariate logistic regression model identified low DBP (defined as less than 70 mmHg) as an individual risk factor for mortality, after adjusting for pulse pressure, CAD, chronic kidney disease, or cerebrovascular disease. Pulse pressure was included as a potential cofounder due to its association with cardiovascular disease and its unique relationship to DBP: decreases in diastolic blood pressure result in increased pulse pressure. Increased pulse pressure has been reported to increase the risk of developing diabetes [11], lead to progression of kidney disease [12], and confer a higher risk for CAD [13]. In this population, pulse pressure had no significant association with mortality after adjusting for DBP and other comorbidities (OR 1.01, 95% CI 1.00–1.02).

Low DBP was not independent of hypertension or diabetes, which suggests that a low DBP may only be harmful as a consequence of antihypertensive therapy or in patients with diabetes. Current literature on aggressive treatment of hypertension among diabetics has failed to show any benefit on mortality [14]. It was startling to learn that 18% of our entire study population had an ambulatory DBP of 60 mmHg or less. Notably, nearly half of the participants had a DBP of 70 or less. These findings underscore a lack of awareness amongst physicians regarding the paucity of evidence showing benefit in aggressive BP lowering and in particular the potential harms associated with it.

Owing to the debatable nature of the clinical significance of the J-curve [15–17], major societal guidelines have not previously given due recognition to the phenomenon. While the seventh report of the Joint National Committee on Prevention, Detection, Evaluation, and Treatment of High Blood Pressure has been the gold standard for nearly a decade [7], it fails to address the question as to the potential harms with lowering blood pressure beyond a certain threshold. Likewise, indications and targets for aggressive blood pressure control have not been well defined. This report also defines the relationship between blood pressure and cardiovascular events as linear and independent of other factors. While this is likely true for patients without existing cardiovascular disease, patients with atherosclerotic CAD and LVH have a more narrow range for which autoregulation of the coronary arterial pressure can occur [18]. Different from the coronary circulation, which is mostly dependent on diastole for perfusion, the cerebral vasculature depends mostly on systolic BP [19] which allows it to tolerate a wider range of mean arterial pressures. Studies have differed in the clinical impact of low DBP on stroke [9, 19, 20]. Our study found that mortality due to low DBP was independent of a history of stroke. These results diverge from prior studies. A post hoc analysis reveals that the prevalence of CAD was 35% in patients with a history of cerebrovascular disease, showing significant overlap in these diseases. This suggests that individualization of BP goals should be tailored to comorbid conditions.

The growing body of evidence for J-shaped relationships between blood pressure and cardiovascular outcomes has led to the revision of guidelines from the European Society of Hypertension [21]. This represents a significant action towards broad recognition of the J-curve. The Joint National Committee is currently in progress in their draft of blood pressure guidelines for their 8th report. One of the questions we hope it will be addressed is how low blood pressure should be reduced.

## 5. Limitations

As with all retrospective studies we were limited by unidentified or incompletely documented potential confounders. The trend towards increased CAD in the group with DBP <70 mmHg, even after matching, poses a particular confounder in the relationship between low DBP and all-cause mortality, though this was accounted for using a multivariate logistical regression model. This bias remains a concern for nearly all trials of BP treatment, that the group which requires the most vigilant treatment could also be the group that possesses the greatest pretreatment cardiovascular risk profile [22]. Hence, we cannot conclude whether the low DBP was due to underlying heart disease, which also confers higher mortality. The study design also does not allow us to find causation, only association between low blood pressure and mortality.

Another limitation is that while this was a large population, it was also a specific population. The Veterans Administration medical record does not routinely record ethnicity as part of patient demographics. While younger veteran groups are more ethnically diverse, World War II and Korean War veterans are more than 88% Caucasian.

Finally, our dataset lacks intervals and averages on the recording of BP, and we are limited to the final reading at the time of data collection. It is not possible to consider trends in BP over time with this limitation. 

## 6. Conclusions

While treatment of hypertension reduces mortality due to cardiovascular events, reduction of DBP below 70 mmHg is associated with increased all-cause mortality in this male predominant study population with significant comorbidities. The relationship between DBP and mortality follows a J-shaped curve. Avoidance of DBP less than 70 mmHg may be advisable in the management of hypertension, although prospective studies are warranted in more representative patient populations. Our findings suggest the need of a shift in paradigm with the guidelines including a minimum as well as a maximum BP target. BP therapeutic goals should also be individualized to the patient's comorbid conditions.

## Figures and Tables

**Table 1:** Characteristics of patients, according to diastolic blood pressure before matching*.

Characteristic	Patients with DBP <70 (*N* = 6175)	Patients with DBP ≥70 (*N* = 8095)	*P* value
Age (yr)	70.7 ± 9.6	64.6 ± 9.5	<0.001
Comorbidities			
Hypertension	4275 (69.2)	5243 (64.8)	<0.001
Diabetes	2201 (35.6)	2006 (24.8)	<0.001
Chronic kidney disease	595 (9.6)	393 (4.9)	<0.001
Cerebrovascular disease	717 (11.6)	607 (7.5)	<0.001
Coronary artery disease	1623 (26.3)	1164 (14.4)	<0.001
Systolic blood pressure	121 ± 15	133 ± 15	<0.001
Diastolic blood pressure	61 ± 6	79 ± 7	<0.001
Pulse pressure	59 ± 14	54 ± 14	<0.001
Number of antihypertensives	1.2 ± 1.2	1.0 ± 1.1	<0.001
Number of antihypertensives			<0.001
0	2182 (35.3)	3483 (43)	
1	1697 (27.5)	2228 (27.5)	
2	1409 (22.8)	1598 (19.7)	
3	684 (11.1)	606 (7.5)	
4	169 (2.7)	156 (1.9)	
5	31 (0.5)	22 (0.3)	
6	3 (0)	2 (0)	
Medications by class			
Beta blocker	1732 (28)	1828 (22.6)	<0.001
Alpha blocker	1110 (18)	885 (10.9)	<0.001
ACE-I/ARB	2531 (47.1)	2569 (36)	<0.001
Calcium channel blocker	1032 (16.7)	957 (11.8)	<0.001
Thiazide diuretic	1402 (22.7)	2000 (24.7)	0.006
Loop diuretic	635 (10.3)	358 (4.4)	<0.001
All-cause mortality	617 (10)	399 (4.9)	<0.001

DBP: diastolic blood pressure; ACE-I: angiotensin converting enzyme inhibitors; ARB: angiotensin receptor blockers.

*Values reported as *N* (%) or means ± SD.

**Table 2:** Characteristics of patients after matching on propensity score*.

Characteristic	Patient with DBP <70 (*N* = 4428)	Patients with DBP ≥70 (*N* = 4428)	*P* value
Age (yr)	68.9 ± 9.7	67.8 ± 9.4	<0.001
Comorbidities			
Hypertension	3001 (67.8)	2940 (66.4)	0.168
Diabetes	1403 (31.7)	1314 (29.7)	0.040
Chronic kidney disease	321 (7.2)	294 (6.6)	0.259
Cerebrovascular disease	425 (9.6)	424 (9.6)	1.000
Coronary artery disease	985 (22.2)	832 (18.8)	<0.001
Systolic blood pressure	125 ± 14	127 ± 12	<0.001
Diastolic blood pressure	62 ± 6	77 ± 5	<0.001
Pulse pressure	62 ± 14	50 ± 12	<0.001
Number of antihypertensives	1.1 ± 1.1	1.1 ± 1.1	0.064
Number of antihypertensives			0.123
0	1693 (38.2)	1722 (38.9)	
1	1193 (26.9)	1263 (28.5)	
2	985 (22.2)	937 (21.2)	
3	433 (9.8)	386 (8.7)	
4	100 (2.3)	107 (2.4)	
5	22 (0.5)	11 (0.2)	
6	2 (0)	2 (0)	
Medications by class			
Beta blocker	1149 (25.9)	1094 (24.7)	0.179
Alpha blocker	681 (15.4)	643 (14.5)	0.257
ACE-I /ARB	1913 (43.2)	1828 (41.3)	0.067
Calcium channel blocker	675 (15.2)	634 (14.3)	0.220
Thiazide diuretic	1029 (23.2)	1038 (23.4)	0.821
Loop diuretic	334 (7.5)	284 (6.4)	0.037
All-cause mortality	367 (8.3)	244 (5.5)	<0.0001

DBP: diastolic blood pressure; ACE-I: angiotensin converting enzyme inhibitors; ARB: angiotensin receptor blockers.

*Values reported as *N* (%) or means ± SD.

**Table 3:** Multivariate analysis of comorbidities on all-cause mortality.

Variable	Odds ratio (95% CI)	*P* value
DBP <70 mmHg	1.34 (1.11–1.61)	0.002
Pulse pressure	1.01 (1.00–1.02)	0.001
Coronary artery disease	1.52 (1.26–1.84)	<0.001
Chronic kidney disease	2.88 (2.28–3.64)	<0.001
Cerebrovascular disease	1.63 (1.28–2.06)	<0.001
Hypertension	0.91 (0.75–1.10)	0.328
Diabetes	1.04 (0.86–1.25)	0.688

DBP: diastolic blood pressure; CI: confidence interval.

## Abbreviations

BP: Blood pressure
CAD: Coronary artery disease
CI: Confidence interval
DBP: Diastolic blood pressure.
